# American Medical Association

**Published:** 1899-04

**Authors:** Wm. B. Atkinson

**Affiliations:** Office of the Permanent Secretary, 1400 Pine St., Philadelphia


### American Medical Association.

OFFICE OF THE PERMANENT SECRETARY,
1400 PINE ST., PHILADELPHIA.

The Fiftieth* Annual Session will be held in Columbus, Ohio, on Tuesday, Wednesday, Thursday and Friday, June 6, 7, 8 and 9, commencing on Tuesday, at 11 a. m.

The delegates shall receive their appointment from permanently organized State medical societies, and such county and district

*No meeting in 1861 and 1862.

medical societies *as are recognized by representation in their respective State societies,* and from the medical departments of the Army, Navy and Marine Hospital Service of the United States.

Each State, county and district medical society entitled to representation shall have the privilege of sending to the Association one delegate for every ten of its regular resident members, and one for every additional fraction of more than half that number; provided, however, that the number of delegates for any particular State, territory, county, city or town shall not exceed the ratio of one in ten of the resident physicians who may have signed the Code of Ethics of the Association.

*Members by Application.*—Members by application shall consist of such members of the State, county and district medical societies entitled to representation in this Association, as shall make application in writing to the treasurer, and accompanying said application with a certificate of good standing, signed by the president and secretary of the society of which they are members, and the amount of the annual membership fee, $5.00. They shall have their names upon the roll, and have all the rights and privileges accorded to permanent members, and shall retain their membership upon the same terms.

At a recent meeting of this Association the following was unanimously adopted:

WHEREAS, The American Medical Association did, at Detroit, in 1892, unanimously resolve to demand of all the medical colleges of the United States the adoption and observance of a standard of requirements of all candidates for the degree of doctor of medicine which should in no manner fall below the minimum standard of the Association of American Medical Colleges; and

WHEREAS, This demand was sent officially by the permanent secretary to the deans of every medical college in the United States, and to every medical journal in the United States; now, therefore, the American Medical Association gives notice that hereafter no professor or other teacher in, nor any graduate of, any medical college in the United States, which shall after January 1, 1899, confer the degree of doctor of medicine or receive such degree on any conditions below the published standard of the Association of American Medical Colleges, will be allowed to register as either delegate or permanent member of this Association.

Each delegate or permanent member, when he registers, is requested to record the name of the section, if any, that he will attend, and in which he will cast his vote for section officers.

Secretaries of medical societies, as above designated, are earnestly requested to forward, at once, lists of their delegates.

Also, that the permanent secretary may be enabled to erase from the roll the names of those who have forfeited their membership, the secretaries are, by special resolution, requested to send to him, annually, a corrected list of the membership of their respective societies.

ORATIONS.

On Medicine—James C. Wilson, Philadelphia.

On Surgery—Floyd W. McRae, Atlanta, Ga.

On State Medicine—Daniel R. Brower, Chicago.

Chairman Committee of Arrangements—Starling Loving, Columbus.

AMENDMENT.

Offered by W. L. Wills, California:

Constitution, Article IV.—Officers. Amend to read: "The following officers, viz: President, four vice-presidents, treasurer, librarian, secretary, assistant secretary, and chairman of committee of arrangements, shall be nominated by a special committee of one member from each State represented at the meeting, and shall be elected annually by the vote on a joint ticket, and shall hold office until their successors are elected."

SECTIONS.

"The chairman of each section shall prepare an address on the recent advancements in the branches belonging to his section, including such suggestions in regard to improvements or methods of work as he may regard important, and present the same, on the first day of the annual meeting, to the section over which he presides. The reading of such address not to occupy more than forty minutes."—By-Laws.

"A member desiring to read a paper before a section should forward the paper, or its title and length (not to exceed twenty minutes in reading) to the secretary of the section, at least one month before the annual meeting at which the paper or report is to be read."—By-Laws.

OFFICERS OF SECTIONS.

*Practice of Medicine.*—Frank Billings, Chicago, chairman; Carroll E. Edson, Denver, secretary.

*Surgery and Anatomy.*—W. J. Mayo, Rochester, Minn., chairman; M. L. Harris, Chicago, secretary.

*Obstetrics and Diseases of Women.*—A. H. Cordier, Kansas City, Mo., chairman; W. D. Haggard, Jr., Nashville, Tenn., secretary.

*Materia Medica, Pharmacy and Therapeutics.*—Thomas H. Stucky, Louisville, Ky., chairman; Leon L. Solomon, Louisville, Ky., secretary.

*Ophthalmology.*—Casey A. Wood, Chicago, chairman; Charles H. Williams, Boston, secretary.

*Laryngology and Otology.*—Emil Mayer, New York, chairman; Christian R. Holmes, Cincinnati, secretary.

*Disease of Children.*—Henry E. Tuley, Louisville, Ky., chairman; L. D. Boogher, St. Louis, secretary.

*Physiology and Dietetics.*—J. Wier, Jr., Owensboro, Ky., chairman; Lee Kahn, Leadville, Colo., secretary.

*Neurology and Medical Jurisprudence.*—Frederick Peterson, New York, chairman; Hugh T. Patrick, Chicago, secretary.

*Cutaneous Medicine and Surgery.*—W. T. Corlett, Cleveland, Ohio, chairman; J. M. Blaine, Denver, Colo., secretary.

*State Medicine.*—Arthur R. Reynolds, Chicago, chairman; W. P. Munn, Denver, Colo., secretary.

*Stomatology.*—George V. I. Brown, Milwaukee, Wis., chairman; Eugene S. Talbot, Chicago, secretary.

WM. B. ATKINSON, Permanent Secretary.

---

The Section on Materia Medica, Pharmacy and Therapeutics of the American Medical Association urges those who desire to read papers in its departments at the Columbus meeting, June 6th to 9th, to send on their names and the title of their papers at once to the Secretary, who is now making up the final program.

LEON L. SOLOMON, Secretary,
323 W. Walnut Street, Louisville, Ky.

